# 7-Ketocholesterol Increases Retinal Microglial Migration, Activation, and Angiogenicity: A Potential Pathogenic Mechanism Underlying Age-related Macular Degeneration

**DOI:** 10.1038/srep09144

**Published:** 2015-03-16

**Authors:** Maanasa Indaram, Wenxin Ma, Lian Zhao, Robert N. Fariss, Ignacio R. Rodriguez, Wai T. Wong

**Affiliations:** 1 Unit on Neuron-Glia Interactions in Retinal Disease, Laboratory of Retinal Cell and Molecular Biology, National Eye institute, National Institutes of Health, Bethesda, MD, USA; 2 Biological Imaging Core, Laboratory of Retinal Cell and Molecular Biology, National Eye institute, National Institutes of Health, Bethesda, MD, USA; 3 Mechanism of Retinal Diseases Section, Laboratory of Retinal Cell and Molecular Biology, National Eye institute, National Institutes of Health, Bethesda, MD, USA

## Abstract

Age-related macular degeneration (AMD) has been associated with both accumulation of lipid and lipid oxidative products, as well as increased neuroinflammatory changes and microglial activation in the outer retina. However, the relationships between these factors are incompletely understood. 7-Ketocholesterol (7KCh) is a cholesterol oxidation product localized to the outer retina with prominent pro-inflammatory effects. To explore the potential relationship between 7KCh and microglial activation, we localized 7KCh and microglia to the outer retina of aged mice and investigated 7KCh effects on retinal microglia in both *in vitro* and *in vivo* systems. We found that retinal microglia demonstrated a prominent chemotropism to 7KCh and readily internalized 7KCh. Sublethal concentrations of 7KCh resulted in microglial activation and polarization to a pro-inflammatory M1 state via NLRP3 inflammasome activation. Microglia exposed to 7KCh reduced expression of neurotrophic growth factors but increased expression of angiogenic factors, transitioning to a more neurotoxic and pro-angiogenic phenotype. Finally, subretinal transplantation of 7KCh-exposed microglia promoted choroidal neovascularization (CNV) relative to control microglia in a Matrigel-CNV model. The interaction of retinal microglia with 7KCh in the aged retina may thus underlie how outer retinal lipid accumulation in intermediate AMD results in neuroinflammation that ultimately drives progression towards advanced AMD.

Age-related macular degeneration (AMD) is a leading cause of irreversible vision loss in older individuals worldwide^1^,^2^, however its pathophysiology remains incompletely understood. The progression of AMD involves a transition from an early or intermediate stage, in which extracellular deposits called drusen accumulate on the inner surface of Bruch's membrane, to an advanced stage featuring photoreceptor and retinal pigment epithelium (RPE) atrophy and/or choroidal neovascularization (CNV), which lead to central vision loss^3^. While the mechanisms driving this progression are unknown, they have been linked to lipid transport and metabolism in the retina as variants in genes involved in these processes have been found to confer increased risk of AMD progression in several genome-wide association studies^4^,^5^,^6^. Additionally, histological studies have demonstrated the accumulation of phospholipids and cholesterol in the Bruch's membrane (BrM)-retinal pigment epithelium (RPE) complex, which increases with aging and AMD stage^7^,^8^,^9^,^10^,^11^. In the highly oxidative environment of the outer retina, these lipids have been noted to undergo conversion to oxidized species^12^, which exert deleterious changes resembling those found in advanced AMD^13^,^14^.

One particular species of oxidized lipid is 7-ketocholesterol, an oxysterol commonly found in oxidized low-density lipoprotein (oxLDL) that is associated with cellular toxicity in vascular endothelial and smooth muscle cells^15^,^16^ as well as in RPE cells^17^. Previous studies have shown that 7KCh is formed by photodamage in the rodent retina via a free radical-mediated mechanism^18^, it localizes to presumed lipoprotein deposits in the non-human primate BrM, choriocapillaris, and RPE layer^19^,^20^, and it accumulates with increasing age, particularly in RPE-capped drusen in aged human eyes^11^. 7KCh is of particular interest in AMD pathobiology^21^ as it has been associated with ocular inflammatory responses and neovascularization *in vivo*^22^,^23^. However, the relevant retinal cell types that 7KCh acts on to drive inflammatory changes in AMD pathogenesis are not completely understood.

Microglia, the resident immune cell of the retina, are responsible for the local modulation of neuroinflammatory change. Microglia in the young, healthy retina are confined to the inner retina, but with aging, these cells migrate to the subretinal space, where they demonstrate increased activation^24^,^25^. This subretinal accumulation of microglia have been associated with disease lesions in AMD histological specimens^26^ and in AMD-relevant animal models^27^, and have therefore been hypothesized to drive photoreceptor and RPE degeneration, as well as choroidal neovascularization^28^,^29^. The mechanisms driving the migration of aging microglia into the outer retina and their subsequent activation have not been well defined.

In this study, we hypothesize that the age-related deposition of 7KCh is related to subretinal microglial recruitment and activation that in turn contributes to progression to neovascular AMD. We evaluated the specific effects that 7KCh exerts on retinal microglial physiology and explored the notion that 7KCh induces pathogenic microglial changes. Our findings described here indicate that 7KCh acts as a chemoattractant capable of inducing the translocation of retinal microglia to the subretinal space. Once there, uptake of 7KCh by microglia can increase microglial activation, M1 polarization, and expression of angiogenic factors in ways that potentiate AMD progression.

## Results

### Age-dependent accumulation of 7KCh in subretinal microglia

**Figure 1:**
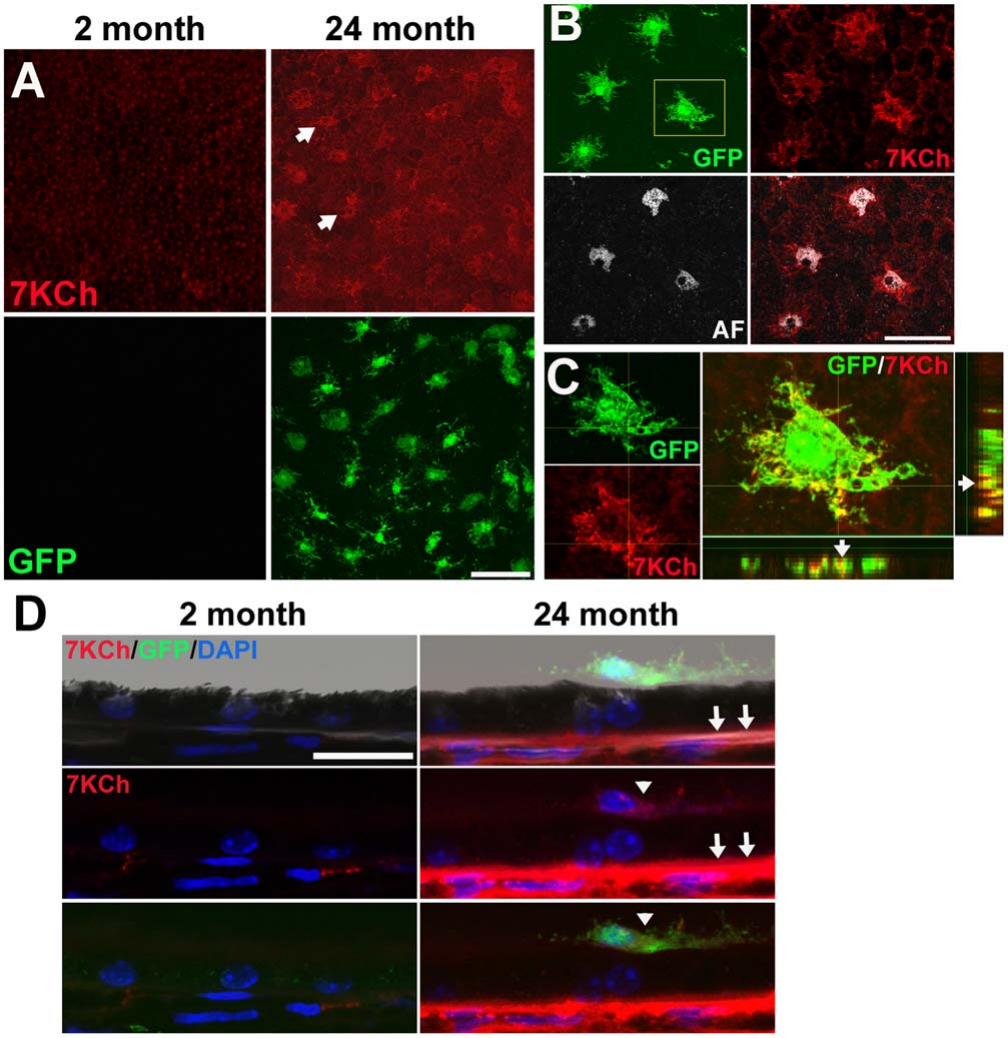
Age-dependent accumulation of 7KCh in the outer retina. (A)(*Upper panels*) Immunohistochemistry for 7KCh in RPE-choroidal flatmounts demonstrates minimal immunopositivity on the apical surface of the RPE monolayer of 2-month old retinas (*left*) but a patchy pattern of staining in 24-month old retinas (*arrows*). (*Lower panels*) Retinal microglia, marked with GFP-expression in CX3CR1^GFP/+^ mice, were absent in the apical RPE surface of 2-month old flatmounts (*left*) but were prevalent in 24-month old retinas (*right*). (B) Patches of 7KCh immunopositivity colocalized to the cytoplasm of GFP-labeled subretinal microglia, and had an intracellular distribution pattern distinct from the perinuclear pattern of autofluorescence (AF) granules. (C) High-resolution confocal imaging of subretinal microglial cell (from inset in B) demonstrates localization of 7KCh immunopositivity (*red*) with GFP signal (*green*) as observed on the orthogonal projections of the confocal image stack, confirming localization of 7KCh to microglia. Scale bars = 50 μm. (D) Brightfield and confocal images of sections of the RPE-choroid complex from 2- and 24-month old animals, demonstrating the presence of 7KCh-immunopositive subretinal microglia (arrowheads), and the increased 7KCh immunpositivity in the sub-RPE layer (arrows) in aged but not young retinas. Scale bar = 20 μm.

To examine the distribution of 7KCh in the outer retina of the mouse, we performed immunohistochemical localization of 7KCh in sclerochoroidal flat-mounts of young (2-month old) and aged (24-month old) CX3CR1^GFP/+^ mice. Little or no immunostaining was detected in samples from young mice, while increased staining with a patchy distribution was evident on the apical RPE surface in aged mice (Fig. 1A). These changes were accompanied by the age-dependent accumulation of GFP-labeled microglia in the subretinal space^30^. The pattern of 7KCh distribution in the cytoplasm of subretinal microglia differed from the perinuclear distribution of a particulate autofluorescence^25^ (Fig. 1B). Colocalization of 7KCh immunopositivity with GFP labeling demonstrated that subretinal 7KCh was concentrated within microglia (Fig. 1C). Immunohistochemistry performed on RPE-choroidal sections confirmed that subretinal microglia were immunopositive for 7KCh and showed that 7KCh immunopositivity also increased in the sub-RPE space in 24-month old retinas relative to 2-month old retinas (Fig. 1D). Experiments performed in the absence of the primary antibody to 7KCh did not detect immunopositivity above background levels (data not shown).

### 7KCh exerts a chemoattractive effect on retinal microglia *in vitro* and *in vivo*

**Figure 2:**
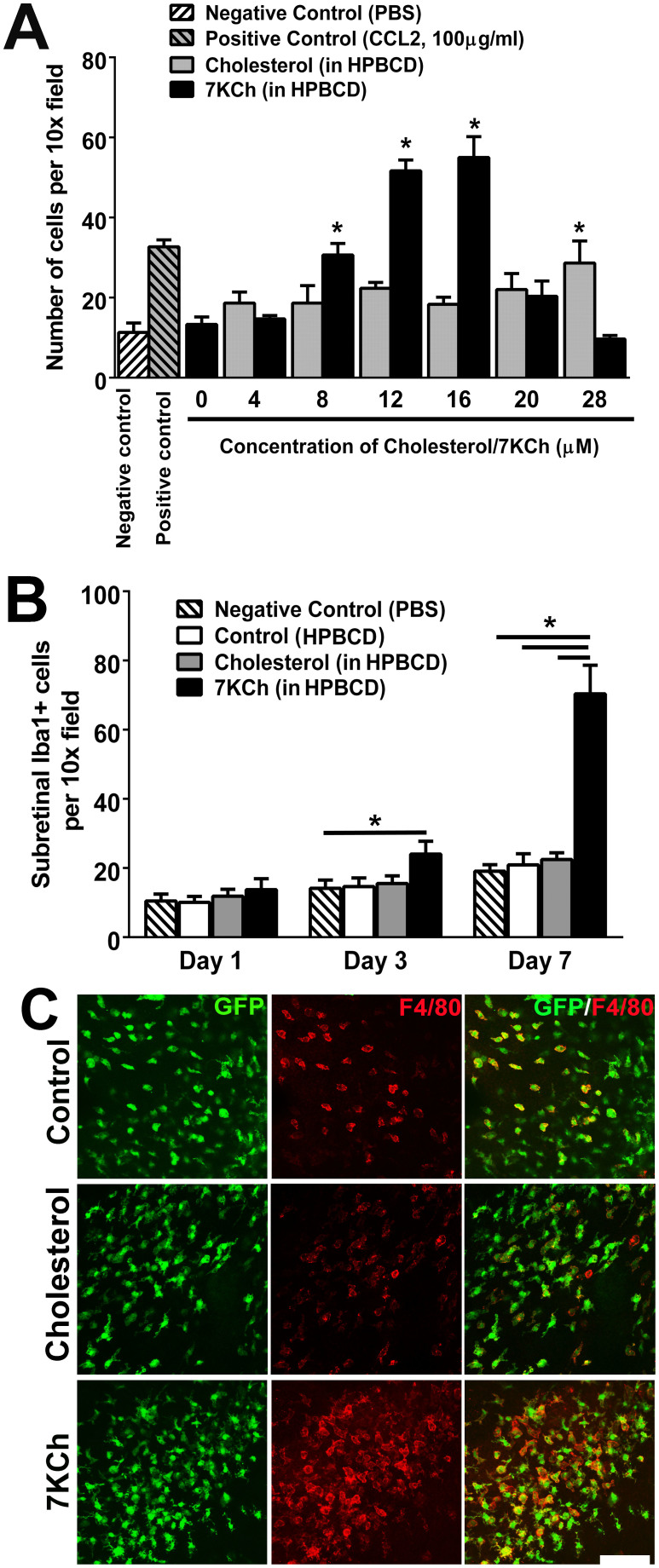
7KCh exerts a chemoattractive effect on retinal microglia *in vitro* and *in vivo*. (A) *In vitro* chemotaxis of cultured mouse retinal microglia to 7KCh was evaluated using a transwell migration assay. Microglial chemotaxis towards 7KCh (in HPBCD) (*black bars*) in the lower chamber increased in a concentration-dependent manner up to 16 μM. A similar response was not observed for unoxidized cholesterol (*gray bars*). Negative and positive controls in the lower chamber (*hatched bars*) included 1× phosphate buffered saline (PBS) and the chemokine CCL2 (100 μg/ml) respectively. (* indicates p < 0.05 relative to control (HPBCD solution only), one-way ANOVA with Dunnett's multiple comparison test, n = 3 biological repeats). (B) *In vivo* migration of retina microglia to 7KCh was assessed following subretinal injection of 7KCh (1.5 μl of 12 μM solution in HPBCD) in 2 month-old C57Bl6 mice. The numbers of subretinal microglia in 10× fields were counted 1, 3, and 7 days following 7KCh injection. At Day 7, the number of subretinal microglia in the area of 7KCh injection was significantly greater than in eyes injected with HPBCD or cholesterol (1.5 μl of 12 μM solution) controls. (* indicates p < 0.05, Kruskal-Wallis test with Dunn's multiple comparison test, n = 13 imaging fields for ≥3 animals per condition.) (C) High-power fields of RPE-choroidal flatmounts showing subretinal microglial accumulation 7 days following subretinal injection into adult CX3CR1^GFP/+^ mice as performed in (B). While subretinal injection of control of HPBCD solution (*upper panels*) or cholesterol (*middle panels*) induced some subretinal microglial accumulation as evidenced by the presence of GFP+ cells, subretinal 7KCh (*lower panels*) induced a significantly greater number of subretinal microglia, the majority of which were F4/80-positive, indicating their activated status. Scale bar = 80 μm.

While the increase in the number of subretinal microglia with age has been well-characterized^25^,^30^, the factors that induce their outward migration from the inner to the outer retina are unknown. To address the hypothesis that the age-dependent accumulation of 7KCh contributes to this altered distribution of retinal microglia, we evaluated the ability of 7KCh to exert a chemoattractive influence on these cells. Using a transwell cell-migration assay, we assessed the ability of cultured murine retinal microglia to migrate towards increasing concentrations of 7KCh in the bottom chamber. We observed that 7KCh in the range of 4–16 μM exerted a prominent dose-dependent chemotaxis of retinal microglia, an effect that was not observed for similarly increasing concentrations of cholesterol (Fig. 2A). This effect however was absent in concentrations of 7KCh greater than 20 μM. We also evaluated this chemoattractive effect *in vivo* by injecting 7KCh into the subretinal space of young (2–3-month old) adult mice and evaluating microglial migration into the outer retina. Counts of Iba1-labelled subretinal microglia revealed that 7KCh injection, relative to cholesterol injection controls, induced significantly increased subretinal migration at 3 and 7 days following injection (Fig. 2B). These recruited microglia were also predominantly immunopositive for F4/80, indicative of their activated status (Fig. 2C).

### Effect of 7KCh on retinal microglial survival and proliferation

**Figure 3:**
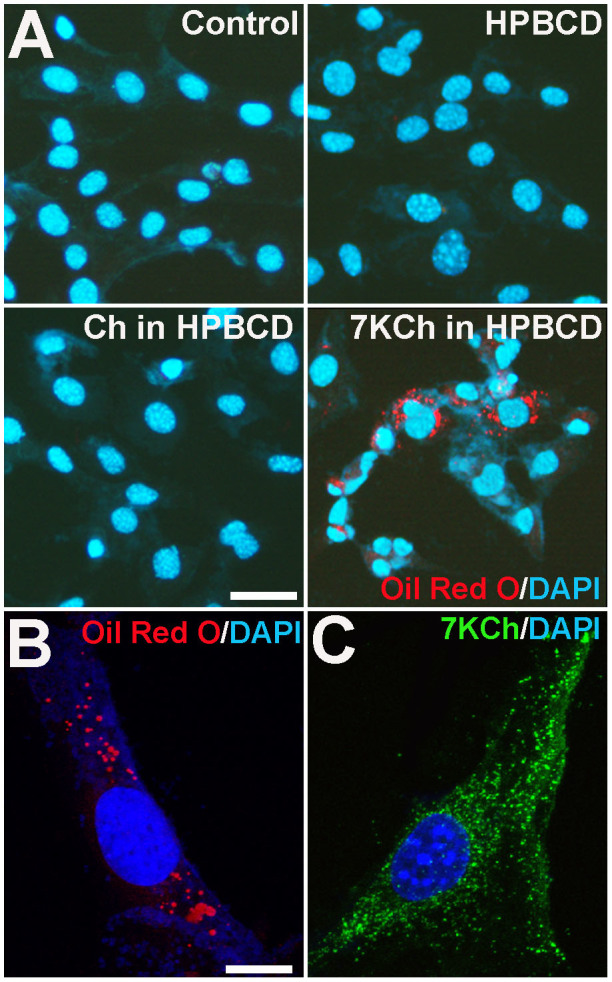
Uptake of extracellular 7KCh by cultured retinal microglia. (A) Cultured retinal microglia were incubated in culture medium alone (*control*), medium containing HPBCD, or supplemented with unoxidized cholesterol (12 μM in HPBCD) or 7KCh (12 μM in HPBCD) for 24 hours, and then fixed and stained with DAPI (*blue*) and Oil Red O (*red*). Cells incubated with 7KCh demonstrated the strongest staining, indicating efficient uptake of extracellular 7KCh by retinal microglia. Scale bar = 25 μm. Cells incubated with 7KCh demonstrated punctate cytoplasmic distribution on both Oil Red O staining (B) and immunostaining for 7KCh (C). Scale bar = 10 μm.

**Figure 4:**
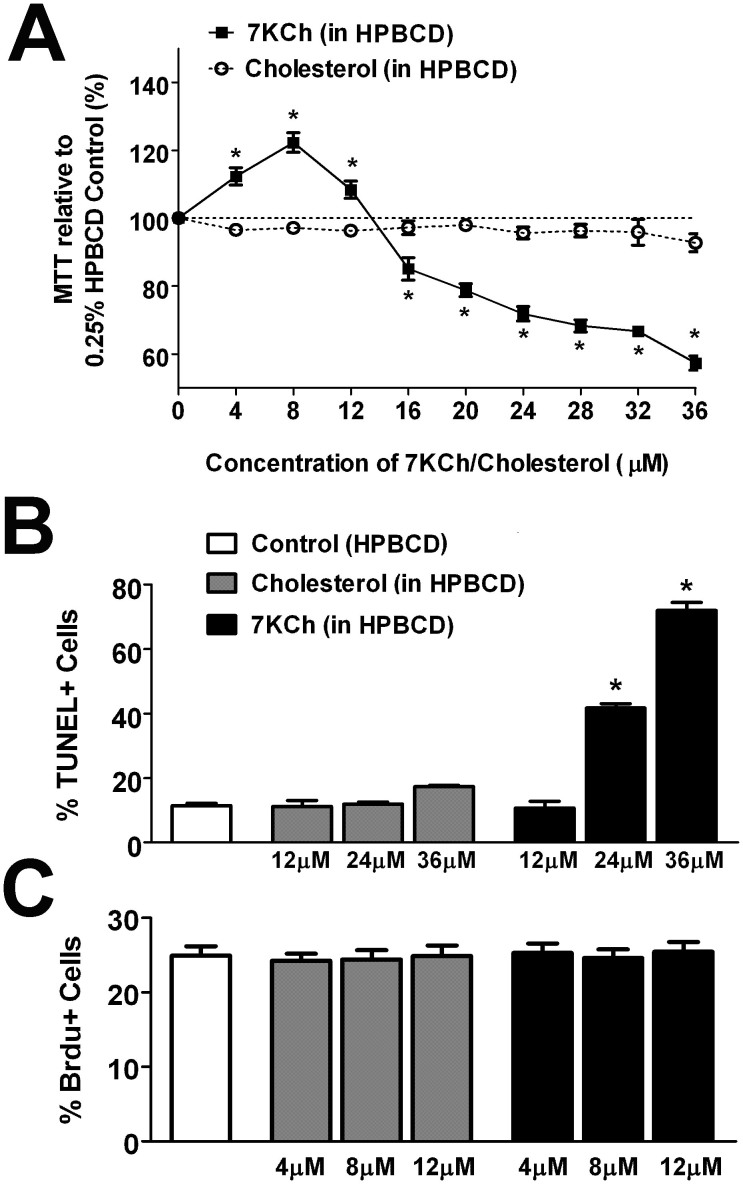
Effect of 7KCh uptake on retinal microglia survival and proliferation. (A) Survival of retinal microglia was assessed following 12 hours incubation in increasing concentrations of 7KCh or cholesterol using a MTT (3-(4,5-Dimethylthiazol-2-yl)-2,5-diphenyltetrazolium bromide) assay. Exposure to concentrations of 7KCh > 16 μM resulted in a concentration-dependent decrease in MTT levels, indicating decreased cellular survival, while exposure to cholesterol (0–36 μM) had no significant effect. (* indicates comparisons to HPBCD-only control for which p < 0.05, 1-way ANOVA with Dunnett's multiple comparison test, n = 6–12 replicates). (B) Incubation of retinal microglial with 7KCh (*black bars*) at concentrations of 24 μM and 36 μM induced significant increases in the proportion of TUNEL+ cells while similar concentrations of cholesterol (*gray bars*) did not (* indicates comparisons to HPBCD-only control for which p < 0.05, Kruskal-Wallis test with Dunn's multiple comparison test, n = 3–9 replicates). (C) Measurements of microglial proliferation, measured by quantifying the proportion of cells incorporating BrdU, were not significantly altered by incubation with either cholesterol or 7KCh (4–12 μM).

To evaluate the effects that 7KCh uptake may exert on retinal microglia physiology, we incubated cultured retinal microglia in culture medium containing 12 μM of 7KCh (12 μM in HPBCD solution), cholesterol (12 μM in HPBCD solution) or HPBCD alone for 12 hours. Microglia incubated with 7KCh demonstrated Oil Red O staining that was markedly more prominent than those incubated with cholesterol (Fig. 3A), indicating a preferential uptake of 7KCh by microglia. Following uptake, Oil Red O staining and 7KCh immunostaining showed a diffuse punctate intracytoplasmic distribution (Fig. 3B, C). We evaluated the effect of the viability of microglia following exposure of increasing concentrations of 7KCh using a tetrazolium dye-based assay. Significant and dose-dependent decreases in cell viability were induced by concentrations of 7KCh above 16 μM (Fig. 4A). This loss of cell viability was likely induced by the activation of apoptosis, as 7KCh also induced a dose-dependent increase in the proportion of TUNEL + apoptotic cells (Fig. 4B). Increasing concentrations of cholesterol (up to 36 μM) had no significant effects on either cell viability or apoptosis, indicating the specific effect of 7KCh on microglia. In the sublethal range of concentrations (4–12 μM), neither 7KCh nor cholesterol exerted any significant effect on microglial proliferation as measured by the proportions of cells incorporating BrdU (Fig. 4C).

### Effect of 7KCh on retinal microglial activation and polarization

**Figure 5:**
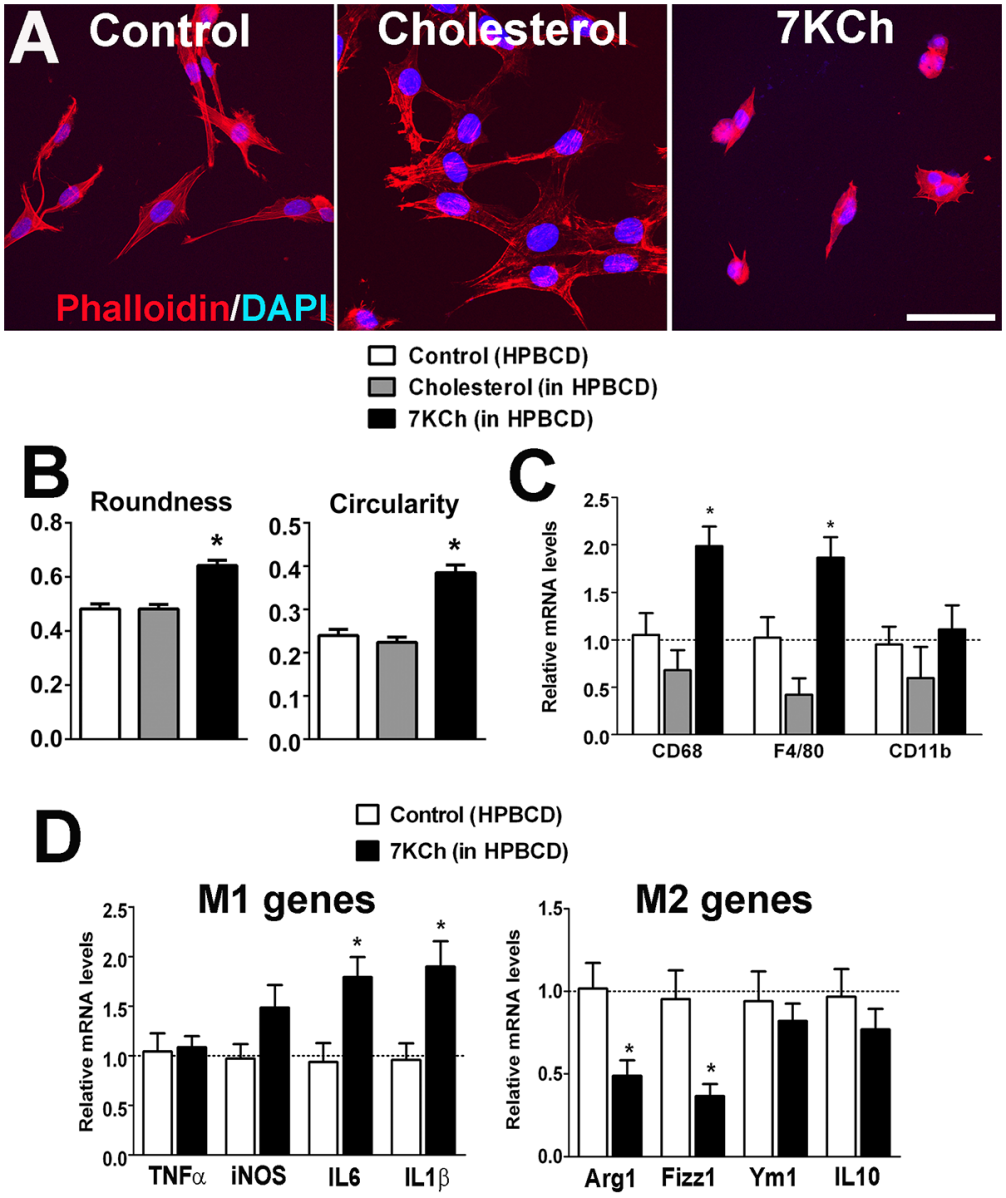
Effect of 7KCh uptake on retinal microglia activation and polarization. (A) Retinal microglia incubated for 12 hours in media containing 0.054% HPBCD (*left*), or supplemented with 12 μM cholesterol (*middle*), or with 12 μM 7KCh (*right*), were fixed and stained with phalloidin (*red*) and DAPI (*blue*). Cells cultured in cholesterol maintained a ramified morphology, characteristic of their resting phenotype, while those exposed to 7KCh have a rounder and more amoeboid morphology, characteristic of an activated state. Scale bar = 50 μm. (B) Computer-assisted morphological analysis of treated cells demonstrated significant increases in the parameters of “roundness” and “circularity” (* indicates comparisons to HPBCD control for which p < 0.05, Kruskal-Wallis test with Dunn's multiple comparison test, n = 126–179 cells). (C) qPCR analyses revealed increased mRNA expression of activation markers CD68 and F4/80 in retinal microglia exposed to 12 μM 7KCh, but not in those exposed to 12 μM cholesterol (* indicates comparisons relative to control (HPBCD only) for which p < 0.05, one-way ANOVA with Dunnett's multiple comparison test, n = 3 biological repeats.) (D) qPCR analyses in retinal microglia following exposure to 12 μM 7KCh showed significant increases in mRNA expression of markers of M1 polarization (IL6 and IL1β) relative to control but significant decreases in markers of M2 polarization (Arg1 and Fizz1), indicating the ability of 7KCh to polarize retinal microglia towards a pro-inflammatory M1 state (* indicates comparisons to HPBCD control for which p < 0.05, unpaired t-test, n = 3).

**Figure 6:**
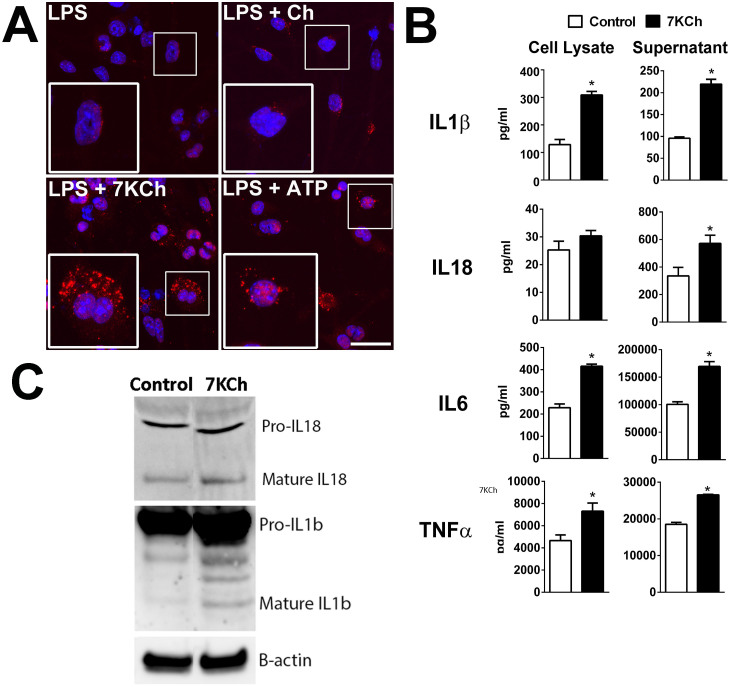
7KCh uptake induces inflammasome activation in LPS-primed retinal microglia. (A) Cultured retinal microglia were primed by exposure to LPS (0.5 μg/ml for 16 hours) and then exposed to cholesterol (12 μM) or 7KCh (12 μM) for 6 hours. Exposure to 5mM of ATP served as a positive control for the induction of inflammasome activation. Cells were fixed and stained with a primary antibody to NLRP3 (*red*) and with DAPI (*blue*). In LPS-primed cells that were either unexposed or exposed to cholesterol, NLRP3 staining was found distributed faintly and diffusely in the cytoplasm with few positive puncta in the perinuclear area (high magnification in inset). In LPS-primed cells that were exposed to 7KCh, a marked increase in the number of NLRP3-positive puncta was observed, indicative of inflammasome activation, as also evident in cells exposed to ATP (positive control). Scale bar = 50 μm. (B) LPS-primed cells exposed to 7KCh demonstrated increased protein expression levels of IL1β, IL6, and TNFα as measured by ELISA in cellular lysates relative to control (LPS-primed cells not exposed to 7KCh). These cells also increased secretion of IL1β, IL18, IL6, and TNFα into the supernatant medium (* indicates comparisons to control for which p < 0.05, unpaired t-test, n ≥ 3). (C) Western blot analyses of cell supernatants of LPS-primed microglia that were exposed to control conditions vs 7KCh for IL18 (*top*) and IL1β (*bottom*) demonstrates increased expression following 7KCh exposure.

As the effect of age-related accumulation of outer retinal lipids may relate to the recruitment and transformation of microglia, we investigated the effects that uptake of sublethal levels of 7KCh may exert on microglial physiology and function. To evaluate the effect of 7KCh uptake on retinal microglial activation, retinal microglia were incubated for 12 hrs in control media, or media containing cholesterol or 7KCh. Analysis of microglial morphology revealed that 7KCh induced a transition from a ramified to a more round and amoeboid morphology typical of microglial activation, a transition not observed in microglia incubated in control medium or medium containing cholesterol (Fig. 5A). Quantitative analysis showed that the morphological measures of “roundness” and “circularity” were significantly increased following 7KCh exposure (Fig. 5B) compared with controls. Retinal microglial mRNA expression of molecular markers of microglial activation, CD68 and F4/80, were also significantly increased following 7KCh exposure (Fig. 5C). 7KCh-exposed microglia showed a polarization towards the proinflammatory M1 state, as evidenced by increases in mRNA expression of M1-associated genes and decreases in M2-associated genes (Fig. 5D). We further investigated if microglial activation by 7KCh involved the formation of the NLRP3 inflammasome. A classic model of NLRP3 inflammasome activation in myeloid cells is a two-step process: a first priming signal, such as that induced by a “pathogen-associated molecular pattern” (PAMP) molecule (e.g. LPS)^31^, followed by a second signal (e.g. P2X7 receptor activation by ATP)^32^, results in the activation and assembly of the inflammasome macromolecular complexes that are evident as cytosolic aggregates/puncta or “speckles”^33^, which lead to the processing of IL1β and IL18 into mature cytokines for secretion^31^. Following priming with LPS, cultured retinal microglia exposed with 7KCh demonstrated an increase in the number of NLRP3-positive cytosolic puncta, indicative of inflammasome activation, which was also induced by ATP exposure, the positive control (Fig. 6A). Protein analysis by ELISA in the cell lysate and conditioned media of 7KCh-exposed microglia indicated increased synthesis and secretion of IL1β and IL18 (Fig. 6B). Quantitative Western blot analysis of mature forms of IL1β and IL18 in supernatants from LPS-primed microglia also demonstrated their increased secretion following 7KCh exposure (increase in 7KCh-exposed cultures relative to control, percentage change ± standard deviation: IL1β: 229.5 ± 20.11%, p = 0.0004; IL18: 170.2 ± 31.0%, p = 0.05, unpaired t test, n = 3 replicates) (Fig. 6C). Protein expression and secretion of proinflammatory cytokines, IL6 and TNFα, were also increased by 7KCh exposure.

### Effect of 7KCh on expression of growth factors by retinal microglia

**Figure 7:**
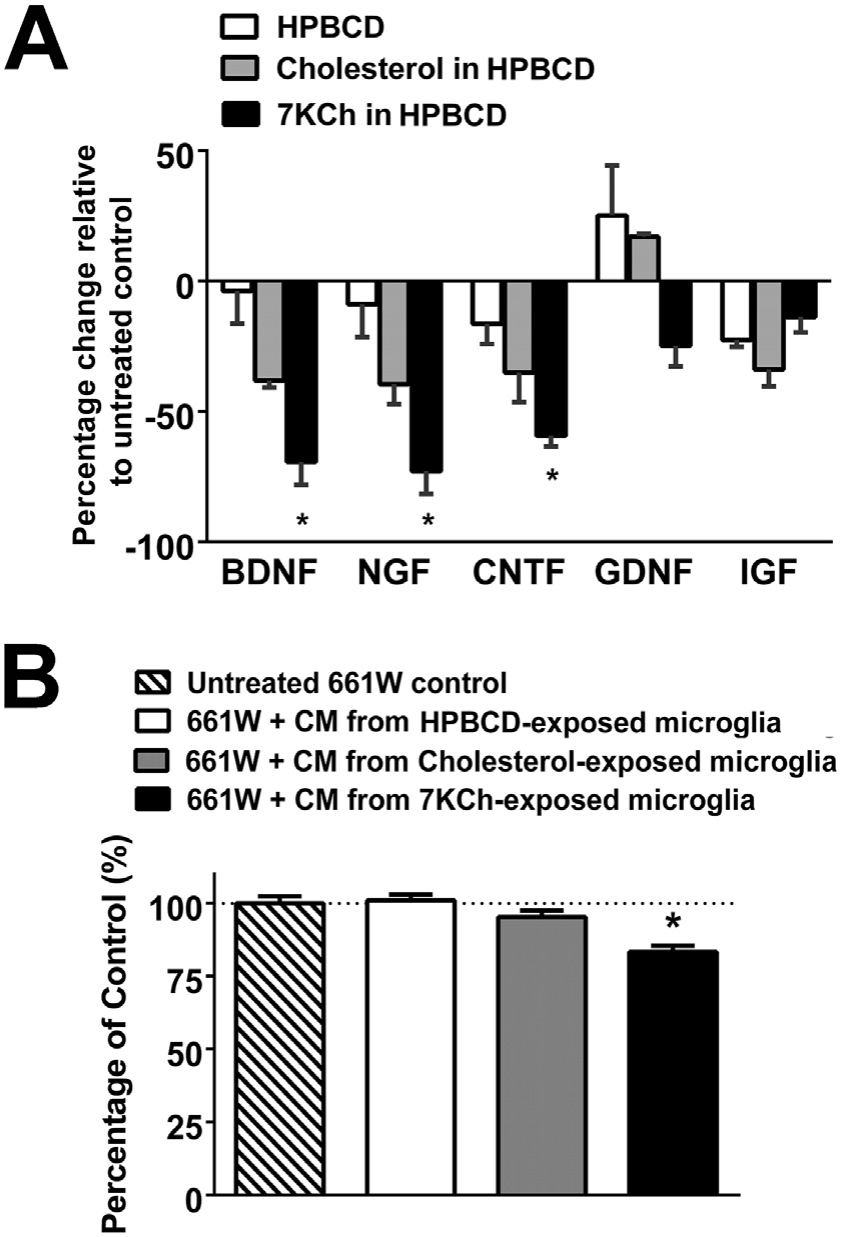
7KCh uptake induces decreased expression of neurotrophic factors in retinal microglia. (A) qPCR analyses in retinal microglia following exposure to control culture media, HPBCD alone, or HPBCD supplemented with either cholesterol (12 μM) or 7KCh (12 μM) for 24 hours. mRNA expression levels of BDNF, NGF, CNTF, but not GBNF and IGF, were significantly reduced in 7KCh-exposed microglia (* indicates comparisons to HPBCD control for which p < 0.05, Kruskal-Wallis test with Dunn's multiple comparisons test, n = 3). (B) 661W cells from a photoreceptor cell line were incubated with control media or conditioned media from microglia cultures that have been exposed to HPBCD only, HPBCD + 12 μM cholesterol, or HPBCD + 12 μM 7KCh. Following 24 hours of incubation, a MTT assay evaluating the survival of 661W cells found significantly decreased photoreceptor cell survival in cultures incubated with conditional media from 7KCh-exposed microglia (indicates comparisons relative to untreated control for which p < 0.05, one-way ANOVA with Dunnett's multiple comparison test, n = 24–48 biological repeats.)

As microglia are a source of growth factor expression^34^ and have been implicated in the provision of support for neuronal survival^35^ and synaptic function^36^ through growth factor-mediated effects, we investigated the effect of 7KCh on microglial expression of growth factors and support of photoreceptors. We found that exposure to 7KCh for 24 hrs significantly decreased microglial mRNA expression of the neurotrophins brain-derived neurotrophic factor (BDNF), nerve growth factor (NGF), and ciliary neurotrophic factor (CNTF) (Fig. 7A), indicating a reduction in the neuroprotective properties in 7KCh-exposed microglia. In support of this, we found that culture of 661W photoreceptors in conditioned media from 7KCh-exposed microglia resulted in a lower survival than photoreceptors cultured with conditioned media from control cells or from cholesterol-exposed microglia (Fig. 7B).

### Effect of 7KCh on the pro-angiogenic properties of retinal microglia

**Figure 8:**
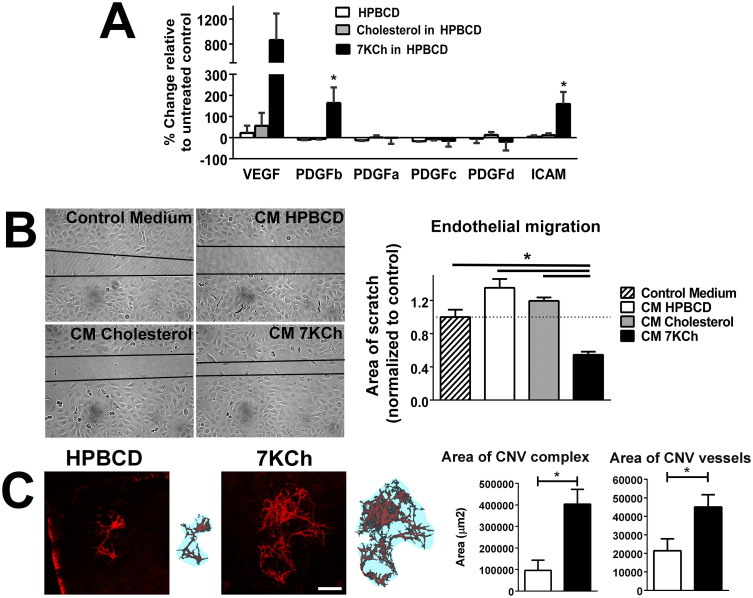
7KCh increases expression of angiogenic factors in retinal microglia and increases the ability of microglia to stimulate angiogenesis *in vitro* and *in vivo*. (A) qPCR analyses in retinal microglia following exposure to control culture media containing HPBCD alone, or HPBCD supplemented with either cholesterol (12 μM) or 7KCh (12 μM) for 48 hours. mRNA expression levels of VEGF, PDGFb, and ICAM were elevated in cultures exposed to 7KCh (*indicates p < 0.05 relative to HPBCD control, 1-way ANOVA, Dunn's multiple comparisons test, n = 3) (B) Endothelial cell scratch assay in which 3B-11 cells were exposed for 12 hrs to (1) control culture medium, or conditioned media from microglia cultures previously exposed to (2) HPBCD (CM HPBCD), (3) 12 μM cholesterol in HPBCD (CM Cholesterol), or (4) 12 μM 7KCh in HPBCD (CM 7KCh). Endothelial cell migration in the presence of conditional media from 7KCh-exposed microglia were significantly increased relative to those in other conditions (*p < 0.05, one-way ANOVA with Tukey's multiple comparison test, n = 12 biological repeats.) (C) The ability of 7KCh to increase the angiogenic potential of retinal microglia *in vivo* was evaluated in a Matrigel model for choroidal neovascularization (CNV). Retinal microglia were exposed to HPBCD or 7KCh (12 μM) in HPBCD for 16 hours and then suspended in growth-factor-deficient Matrigel. Microglia (2–5 × 10^5^ cells) in 1.6 μl of Matrigel was injected into the subretinal space of 3-month old C57Bl6 mice; CNV outgrowth was evaluated in RPE flatmounts 7 days later. Representative examples of CNV membranes in each experimental condition are shown (*left panels, in red*). Scale bar = 300 μm. The retinal area covered by the CNV complex (*blue area* in *right panels*), as well as the area occupied by CNV vessels themselves (*red area* in *right panels*), were significantly increased in the presence of 7KCh-exposed microglia relative to control (*p < 0.05, unpaired t-test with Welch's correction, n = 8–11 animals in each group).

As the accumulation of peroxidized lipids in aged human Bruch's membrane and in animal models has been associated with choroidal neovascularization (CNV), we investigated if 7KCh exposure increased the proangiogenic properties of retinal microglia *in vitro* and *in vivo*. We found that exposure of 7KCh to cultured microglia for 48 hrs increased the mRNA expression of proangiogenic molecules VEGF, PDGFb, and ICAM (Fig. 8A). Conditioned medium from cultured microglia exposed to 7KCh was also able to promote migration of endothelial cells in an endothelial cell “scratch” assay^37^ to a significantly larger extent than conditioned media from cholesterol-exposed microglial or from control microglia (Fig. 8B). To evaluate the ability of treated microglia to promote angiogenesis in vivo, we transplanted control and 7KCh-exposed microglia into the subretinal space in the context of a Matrigel-induced CNV model^38^. Measurements of both the area involved by CNV and the area of the CNV vessels themselves demonstrated that the presence of 7KCh-exposed microglia significantly promoted CNV formation in terms of the size and extent of the CNV complex relative to control microglia (Fig. 8C).

## Discussion

The search for etiologic factors driving the progression of AMD has identified outer retinal lipid accumulation as an alteration in early and intermediate AMD that may be central to progression to late AMD^39^. This has been characterized as age-related deposition of lipid in BrM^10^,^40^,^41^ and the formation of intermediate AMD-associated lesions, such as drusen, basal linear deposits^42^,^43^, and subretinal pseudodrusen^44^, which are lipid-rich in their compositions. These lipid deposits likely originate at least in part from polarized secretion from RPE cells^45^. Over time, these lipid deposits accumulate in the highly metabolically active environment of the outer retina where they undergo oxidative change, forming biologically-active oxidized compounds^12^,^13^,^22^.

One such class of compounds are oxysterols, which are products of cholesterol oxidative metabolism that have been associated with multiple chronic diseases involving inflammation and oxidative damage^46^. 7KCh, the most abundant oxysterol found in the retina, has been implicated in chronic inflammation in the retina^21^. Detected in both the aged primate and human retina-RPE complex^11^,^19^, 7KCh is produced from cholesterol via a free radical-mediated mechanism likely catalyzed by iron, which also increases with aging in the outer retina^47^. The major pathologic effects of 7KCh have been related to inflammation and cell death in cell types such as vascular endothelial cells^48^,^49^, vascular smooth muscle cells^50^,^51^, monocytes/macrophages^52^,^53^, as well as RPE cells^17^,^54^. As retinal microglia, the resident immune cell of the retina, demonstrate age-related changes in their distribution into the subretinal space^25^,^30^ and have themselves been associated with AMD pathogenesis^24^,^26^,^27^,^28^, we investigated the connection between 7KCh accumulation in the outer retina and pathological changes in retinal microglia observed in AMD.

We found in our immunohistochemical studies that 7KCh is elevated in the the subretinal and sub-RPE space of aged mice relative to young mice. Interestingly, we found that in both *in vitro* and *in vivo* studies 7KCh exerts a prominent dose-dependent chemoattractive effect on retinal microglia, an effect that was specific to 7KCh and absent for non-oxidized cholesterol of a similar concentration range. Subretinal injection of 7KCh, but not cholesterol, was able to induce markedly increased number of Iba1 + microglia into the outer retina. This finding indicates that 7KCh accumulating in the aging outer retina may signal at a distance to microglia in the inner retina to induce their migration into the outer retina. It is possible that additional immune cells may also be recruited by 7KCh buildup such as macrophages resident in the adjacent choroid^55^, or systemic monocytes from the general circulation. In support of this notion, oxysterols, in the context of atherosclerosis and tumorigenesis, have also been previously implicated in disease pathogenesis via recruitment of macrophages and neutrophils, possibly via molecular mechanisms involving chemokine signaling (e.g. CCL2, CXCR2)^56^,^57^. As such, 7KCh accumulation with aging may constitute an initiating step in the age-related alteration of the immune environment of the outer retina.

We observed that retinal microglia *in vitro* have a high affinity for 7KCh and in fact internalize 7KCh readily from the culture medium. We also found that subretinal microglia in aged mice were significantly immunopositive for 7KCh, indicating *in vivo* uptake. Cytotoxic effects of 7KCh on retinal microglia *in vitro* were detected for concentrations in excess of 16 μM, similar to observations in ARPE19 RPE cells (>20 μM)^54^. At sublethal concentrations, 7KCh exerted a prominent activation of retinal microglia as assessed by morphology, proinflammatory gene expression, and cytokine secretion. In cell types such as smooth muscle cells, endothelial cells, and macrophages, 7KCh induces proinflammatory gene expression via increased reactive oxygen species (ROS) production and nuclear factor *κ*B (NF*κ*B) activation^58^,^59^,^60^. In retinal microglia, we found that NLRP3 inflammasome activation appears to be involved in this response, driving the increased expression and secretion of IL1β and IL18. Although NLRP3 inflammasome activation in RPE cells has been implicated in AMD pathogenesis^61^,^62^,^63^, a parallel activation in retinal microglia, induced by 7KCh exposure, may constitute an additional contributory factor.

We observed that 7KCh had effects on retinal microglial gene expression, decreasing neurotrophic growth factor expression and conversely increasing cytokine and angiogenic factor expression. As the accumulation of retinal microglia in the outer retina has been associated with the atrophic form of AMD, in which photoreceptor and RPE degeneration occur^26^,^27^,^64^, as well as the neovascular form of AMD, in which CNV develops^28^,^65^, we hypothesized that 7KCh accumulation observed in aging animal models may induce these microglial alterations that are contributing to these processes. One caveat to note here is that while subretinal microglia have been described in human retinal sections^24^,^26^,^27^, quantitative analysis of microglia distribution across the retina has not to our knowledge been performed, and as such, comparisons of findings involving microglia in humans and mouse models are qualitative in nature. We found that 7KCh-exposure was indeed able to amplify the neurotoxicity of retinal microglia to 661W, likely induced by both cytotoxic inflammatory cytokine up-regulation and growth factor down-regulation in exposed microglia. Also, we found that 7KCh-exposure was able to augment the angiogenicity of retinal microglia in both *in vitro* and *in vivo* assays of angiogenesis. Taken together, 7KCh accumulation in the outer retina can therefore not only potentiate subretinal microglia recruitment but also transform their phenotypes to favor retinal degeneration and neovascularization in ways relevant to AMD pathogenesis.

While the RPE monolayer defines the outer blood-retinal barrier and regulates transport into and out of the retina, AMD-related accumulation of lipid deposits has been characterized on either side of the RPE layer in both subretinal and sub-RPE compartments^8^,^44^. Analogously, innate immune cells in the forms of microglia from within the retina^27^,^64^ and circulating monocytes from outside of the retina proper^66^,^67^ have been associated with the RPE layer in AMD-relevant animal models. In addition, RPE-associated immune cells have been reported to traffic across the RPE layer in different pathological contexts^68^,^69^. As a result, it is currently difficult to ascribe AMD-related pathogenicity exclusively to either microglia or monocytes, particularly with respect to CNV which itself often extends across the RPE layer. While the locus of CNV may be conceptualized as being localized beneath the RPE (Type 1) or extending through the RPE (Type 2), actual clinical patterns include combined patterns and even a continuum of patterns^70^,^71^. The subtype of CNV known as retinal angiomatous proliferation (RAP) is a particular illustration of cross-boundary CNV extension as it connects the retinal and choroidal circulations, traversing across the RPE layer^72^. It is likely that age-related changes in lipid deposition on either side of the RPE layer can exert both short- and long-range influences on immune cell recruitment and activation in and around the RPE layer that together alters immune regulation in the retina in ways that culminate in pathological consequences.

In conclusion, in demonstrating and characterizing the effects of 7KCh on retinal microglia, our findings connect two significant associations in AMD pathobiology: 1) the accumulation of oxidized lipids, particularly 7KCh, in the aged and AMD retina, and 2) the recruitment and activation of microglia in the outer retina, which alter the immune environment and potentiate the progression of AMD. The results here indicate that initial 7KCh accumulation in drusen and other retinal lipid-containing extracellular deposits may induce microglial translocation into the outer retina. Uptake of 7KCh by microglia in turn results in activation and physiological changes in microglia that alters the immune environment favoring AMD progression. In this light, the molecular mechanisms underlying 7KCh-mediated effects on retinal microglia may constitute potential therapeutic targets for the treatment and prevention of AMD.

## Methods

### Experimental animals

Heterozygous CX3CR1^GFP/+^ transgenic animals were created by breeding CX3CR1^GFP/GFP^ mice^73^ to C57BL/6J mice (The Jackson Laboratory, Bar Harbor, ME). Both young (3–4 month old) and aged (21–24 month old) wild type C57BL/6J and CX3CR1^+/GFP^ mice were used. Animals were genotyped and confirmed to lack the rd8 mutation^74^. Mice were bred and housed in a National Institutes of Health animal facility in a temperature and light controlled environment with a 12-hour day-light cycle. Experimental protocols, approved by a local Institutional Animal Care and Use Committee, adhered to the Association for Research in Vision and Ophthalmology statement for animal use. Experiments were carried out in accordance with these guidelines and regulations.

### Chemicals and reagents

Cholesterol and 7KCh were purchased from Steraloids, Inc (Newport, RI). Hydroxypropyl-β-cyclodextrin (HPBCD) was purchased from Sigma-Aldrich (St. Louis, MO). HPBCD-7KCh solutions were prepared as previously described^19^ to generate a stock of 10 mM 7KCh solution in 45% HPBCD. In all experiments involving the use of 7KCh, 7KCh was administetered in HPBCD. MCP1 recombination protein was purchased from R&D (Minneapolis, MN).

### Cell culture

Retinal microglia were isolated from postnatal day (P) 5 to 20 C57BL/6J wild type mice and heterozygous CX3CR1^+/GFP^ transgenic mice as previously described^24^. Briefly, retinal cells were dissociated by digestion in 2% papain, followed by trituration and centrifugation. Resuspended cells were transferred into 75-cm^2^ flasks containing Dulbecco's Modified Eagle Medium (DMEM): Nutrient Mixture F-12 media with 10% fetal bovine serum (FBS) (Gibco, Carlsbad, CA, USA) and nonessential amino acids solution (Sigma, St. Louis, MO, USA). Following overnight culture, the medium and any floating cells were discarded and replaced with fresh medium. When the cells grow to confluence, culture flasks were shaken gently to detach microglial cells, which were subcultured in new 75-cm^2^ flasks or 6-well plates. When the microglial cultures were 60%–70% confluent, they were harvested for subsequent experiments. Cultures of the photoreceptor cell line 661W (gift from Dr. Muayyad Al-Ubaidi, University of Oklahoma Health Sciences Center, OK) were prepared as previously described^75^.

### Immunohistochemistry and cell labeling

Retinal and sclerochoroidal flat mounts were prepared as described previously^76^. Briefly, enucleated eyes were dissected to form posterior segment eye-cups which were then fixed in 4% paraformaldehyde in phosphate buffered saline (PBS) for 2–4 hours at 4°C. After washing with PBS, the retina was dissected free, transferred into 1% Triton-X100 (Sigma, St. Louis, MO) for 1 hour at room temperature, and then incubated in blocking reagent (Roche, Indianopolis, IN, USA) for 30 minutes on a shaker. Cultured cells were similarly fixed and permeabilized with 4% paraformaldehyde, 0.25% Triton-X100 in PBS. Sclerochoroidal flat mounts and cultured cells were immunostained with primary antibodies to the following antigens: anti-ionized calcium binding adaptor molecule-1 (Iba1) (1:500, Wako, Richmond, VA), F4/80 (1:200; Abd Serotec, Raleigh, NC), phalloidin (Alexa-488 or -633-conjugated, 1:150; Invitrogen, Carlsbad, CA, USA, #A22284, 1:100), NLRP3 (1:100, Novus Biologicals, Littleton, CO), and 7KCh (1:100; Clone #35A, Japan Institute for the Control of Aging, Shizuoka, Japan). Secondary antibodies, conjugated to Alexa-488, Alexa-568, or Alexa-633 (Invitrogen), were added at a 1:200 dilution and incubated for 1–2 hours. 4′,6-diamidino-2-phenylindole (DAPI, Molecular Probes/Invitrogen, Cat# D1306) was used to label cellular nuclei. Oil Red O (Sigma) was used to stain intracellular lipid in fixed microglial cells. Fixed cells were incubated with 1 ml of 60% isopropanol for 5 min, and then dried. One ml of Oil Red O solution (diluted 3:5 with H_2_O and 0.3% Oil Red O stock in isopropanol) was added and incubated for 5–10 min, before washing with PBS.

### Microglial chemotaxis assay

Microglial chemotaxis was evaluated using a 48-well modified Boyden chemotaxis assay (Neuroprobe, Gaithersburg, MD). A 5-mm polycarbonate filter (Neuroprobe, Gaithersburg, MD) separated the top and bottom chamber compartments. Cultured retinal microglia cells were harvested with 0.25% trypsin-EDTA and re-suspended in 5% heat-inactivated FBS in DMEM to a concentration of 2.0 × 10^5^ cells/ml. A total of 1.0 × 10^4^ cells in 50 μl was added to the top chamber while a 50 uL volume consisting of either of 1) 7KCh (12 μM in HPBCD solution), 2) cholesterol (12 μM in HPBCD solution), 3) control media (HPBCD solution), 4) positive control of the chemokine CCL2 (100 μg/ml in PBS), or 5) negative control (1× PBS), was added to the bottom chamber. The entire chamber was then incubated for two hours at 37°C after which the bottom portion of the membrane filter (containing adherent cells migrating towards the bottom chamber) was fixed in 4% paraformaldehyde in PBS for 30 minutes and stained for cell nuclei with DAPI (Vector Labs, CA). Images of stained cells were visualized using an epifluorescence microscope at 10× magnification and the cell numbers counted using an automated cell-counting algorithm (ImageJ, NIH, Bethesda, MD).

### Assays of cell viability, proliferation and apoptosis

Survival rates of retinal microglia and photoreceptor 661W cells cultured under the different conditions were assessed using a dimethylthiazol-diphenyltetrazolium bromide (MTT) assay kit (Trevigen, Gaithersburg, MD) according to the manufacturer's directions. Cell proliferation of microglia was assessed by bromodeoxyuridine (BrdU) labeling. Cultured cells were incubated with 10 μg/ml BrdU (Invitrogen, CA) for 45 minutes at 37°C. They were then fixed with 4% paraformaldehyde in PBS for 30 minutes, permeabilized with 1% Triton X-100 in PBS for 30 minutes and PBST:HCL(5N, 1:1) for 1 hr at room temperature, incubated in 2% blocking solution (Rodeo™ Blocker, USB, OH) for 30 minutes, and then stained with anti-BrdU antibody (1:200, Developmental Studies Hybridoma Bank, Iowa City, IA) overnight followed by an anti-mouse secondary Alexa-633 antibody (1:200, Invitrogen, CA) for two hours. BrdU-positive labeling was quantified using the ImageJ (National Institutes of Health, MD) automated cell counting program on images of representative 20× fields obtained from epifluorescence microscopy. Apoptosis of microglia was measured by terminal deoxynucleotidyl transferase (TDT) dUTP nick end labeling (TUNEL) assay according to the manufacturer's directions (Roche, Nutley, NJ). TUNEL-positive cells were quantified in 20× objective imaging fields using automated cell counting algorithms (ImageJ).

### Morphological analysis of cultured microglia

Cultured retinal microglia, following exposure to control conditions, cholesterol (12 μM), or 7KCh (12 μM) were fixed, permeabilized, and immunostained with Alexa-633 conjugated phalloidin (1:150, Invitrogen, CA) to reveal their cellular morphology. Cells were imaged with confocal microscopy using ImageJ software. Two morphological parameters were quantitated: “roundness” which is defined as 4*(Area)/π(Major axis)^2^, “circularity” which is defined as 4 π (Area)/(perimeter)^2^. On these two measures, a perfect circle will have a value of 1.0 while more elongated shapes will have values < 1.

### Quantitative reverse transcription polymerase chain reaction (qRT PCR)

Cultured cells were lysed by trituration and homogenized using QIAshredder spin columns (Qiagen, Valencia, CA). Total RNA was isolated using the RNeasy Mini kit (Qiagen) according to the manufacturer's specifications. First-strand cDNA synthesis from mRNA was performed using qScript cDNA SuperMix (Quanta Biosciences, Gaithersburg, MD) using oligo-dT as primer. qRT-PCR was performed using a SYBR green RT-PCR kit (Affymetrix, Cleveland, Ohio), using the 7900HT Fast Real-Time PCR System (Applied Biosystems, Carlsbad, CA, USA) under the following conditions: denaturation at 95°C for 5 minutes, followed by 40 cycles of 95°C for 10 seconds and then 60°C for 45 seconds. Threshold cycle (CT) values were calculated, and expressed as fold induction determined using the comparative CT (2^−ΔΔCT^) method. Glyceraldehyde-3-phosphate dehydrogenase (GAPDH) was used as an internal control. Oligonucleotides used are listed in Supplementary Table 1.

### Analysis of protein expression

Cultured microglial cells were primed by exposure to lipopolysaccharide (LPS) (0.5 μg/ml for 16 hours) and then exposed to either control conditions or 7KCh (12 μM) for 6–12 hours. Following exposure, the supernatants were collected and the cells lysed by trituration in RIPA buffer (Sigma, St. Louis, MO) containing 1:100 proteinase inhibitor (Calbiochem, Gibbstown, NJ). The levels of IL1β, IL18, IL6, and TNFα protein in both the cell lysates and supernatants were assayed with a commercial chemiluminscence-based ELISA (Searchlight, Aushon Biosystems, Billerica, MA). Levels of IL1β and IL18 in cell supernatants were also evaluated by Western blotting. Briefly, supernatants were mixed with SDS loading buffer, boiled, run on a 12% NuPAGE Bis-Tris gel, and then transferred into a nitrocellulose membrane (Invitrogen). The blot was incubated with primary antibodies to IL1β (AF-401-NA, 1:1000, R&D) and IL18 (D046-3, 1:200, MBL, Woburn, MA) overnight at 4°C, followed by incubation with HRP-conjugated anti-rat (1:3000, Cell Signaling, Danvers, MA) or anti-goat (1:3000, Santa Cruz, Dallas, TX) secondary antibodies for 2 h at room temperature. A conjugated primary antibody to beta-actin (HRP-anti B-actin, A3854, 1:25000, Sigma) was used as internal control. The blots were scanned (LAS-3000 Imager, Fujifilm, Tokyo, Japan) and analyzed using image analysis software (ImageJ). At least 3 replicates of each experiment were obtained.

### Endothelial cell scratch wound assay

An endothelial cell line, 3B-11, (CRL-2160, ATCC, Manassas, VA) were used to monitor endothelial migration in a scratch-wound assay^77^. Briefly, endothelial cells were grown to confluence on 6-well tissue culture plates whereupon a scratch wound of uniform width was made with a sterile 200-ml pipette tip through the center of the well. After wounding, the cultures were washed 3 times with serum-free DMEM medium. Control medium or conditioned medium from microglial cultures exposed to HPBCD solution, cholesterol (12 μM) in HPBCD, or 7KCh (12 μM) in HPBCD were then added to each well. After 12 hours, the “wound” was photographed on an inverted microscope and the area of a standard length of the “wound” quantified using ImageJ image analysis software.

### Subretinal injection of 7KCh

To evaluate *in vivo* microglial responses to the presence of 7KCh in the outer retina, subretinal injections of 1.5 μl volume containing either 1) control solution (0.054% HPBCD in PBS), 2) cholesterol (12 μM + 0.054% HPBCD in PBS), 3) 7KCh (12 μM + 0.054% HPBCD in PBS), or 4) a negative control (PBS only) were performed in 2 month-old adult C57Bl6 WT or CX3CR1^GFP/+^ animals as previously described^78^. Animals were sacrificed at 1, 3, and 7 days (n = 3 animals per time point) post-procedure and sclerochoroidal flat-mounts prepared. Microglial migration into the subretinal space was visualized with either immunhistochemical staining for Iba1 or F4/80. Images of subretinal microglia were captured using a confocal microscope in sclerochoroidal flat mounts and microglial cell counts obtained.

### Subretinal injection of retinal microglia

Cultured retinal microglia were exposed to either media containing 7KCh (12 μM) or control media for 16 hours and suspended in growth factor-reduced Matrigel (BD Biosciences, Bedford, MA) at a 1:1 volume ratio. A volume of 1.6 μl of microglia-containing Matrigel (≈2 × 10^5^ cells) was injected into the subretinal space of 3–4 month-old adult C57Bl6 mice. Following 7 days after subretinal injection, the structure of the choroidal neovascular (CNV) membrane, was assessed by intracardiac lipophilic dye perfusion as previously described^28^,^79^. Briefly, a perfusate of 1, 1′-Dioctadecyi-3, 3, 39, 39-tetramethylindocarbocyanine perchlorate (DiI, Sigma-Aldrich, St.Louis, MO) was prepared diluting a stock solution (6 mg/mL in 100% ethanol) in a diluent of PBS containing 5% glucose in a 1:250 ratio. Experimental animals were euthanized by overdose of solution containing ketamine (300 mg/kg) and xylazine (30 mg/kg) and perfused intracardially with the DiI perfusate at a rate of 1 to 2 mL/min. Eyes were then harvested and sclerochoroidal whole-mounts of entire posterior segments prepared. The injection entry site and the area covered by the subretinal Matrigel-cell mixture were located on the whole-mounted tissue, and the resulting CNV membrane photographed with an epifluorescence microscope and/or confocal microscopy (SP2; Leica, Exton, PA). The area subtended by the branched CNV membrane (i.e. area of the CNV complex) and the area occupied by the CNV vessels (i.e. are of CNV vessels) were quantified using image analysis software (NIH Image J).

### Statistical analysis

Statistical analyses were performed using statistical software (GraphPad, version 5.00, San Diego, CA, USA). Comparisons of 2 data groups were performed using an unpaired t-test (with the null hypothesis that the population means are equal), while 3 or more data groups were performed using 1-way analyses of variance or non-parametric Kruskal-Wallace test (with the null hypothesis that the samples come from populations with the same distribution). Multiple comparisons between pairs of group means were performed with the Tukey, Dunn or Dunnett multiple comparison tests. In all graphical representations, the error bars indicate standard error of the mean.

## Author Contributions

M.I., W.M., I.R.R. and W.T.W. conceived and designed experiments. M.I., W.M. and L.Z. performed the experiments. M.I., W.M., L.Z. and W.T.W. analyzed the data. R.N.F., I.R.R. and W.T.W. contributed reagents/materials/analysis tools. M.I., W.M., L.Z., R.N.F., I.R.R. and W.T.W. wrote the paper. All authors reviewed the manuscript.
